# Did you cite your sources? Why bibliography matters

**DOI:** 10.3389/fmed.2026.1947650

**Published:** 2026-10-27

**Authors:** Sarah M. Cohen, Simcha Yagel

**Affiliations:** Division of Obstetrics and Gynecology, Hadassah Medical Center and the Faculty of Medicine of the Hebrew University of Jerusalem, Jerusalem, Israel

**Keywords:** AI in publishing, bibliography, citations, LLMs in scientific writing, scholarly writing and publishing, scientific publication

## Abstract

The burgeoning artificial intelligence ecosystem has changed every facet of the landscape of medical research, including the dissemination of results by writing and publishing research papers. The presence of hallucinated (fabricated, inaccurate, or incomplete) references in your bibliography calls into question the basis and validity of your work. In this Perspective, we reiterate the imperative of properly curating the reference list to support each standard section of a research article.

## Perspective

“If I have seen further, it is by standing on the shoulders of giants,” (1) Isaac Newton's well-known aphorism of scientific humility, continues to resonate with researchers today because it will always be true, no less so because he himself was referencing medieval philosopher Bernard of Chartres (2). No advance in any field is ever solely the product of one mind working in isolation. Progress is cumulative, resting on the work of previous generations of researchers. To recognize and acknowledge that earlier work as the basis of your own, even when your work refutes and disproves what has come before, is an essential part of scientific writing.

It is no secret that with the help of a burgeoning artificial intelligence ecosystem, research papers that formerly demanded hours or weeks of a researcher's time can emerge in minutes, seemingly full-grown. Large language models (LLMs) will also helpfully provide sources for their statements, even offering to format them according to your target journal. Upon review, however, these proffered sources may be “hallucinated”; they may be inaccurate, incomplete, or fabricated. The authors’ names, article titles, or journal names may closely resemble or mimic those of authentic publications, but a search for the citation in a database like PubMed or a reference manager will reveal that the publication does not exist. Researchers tested (3) the prevalence of fabricated references in texts produced by ChatGPT-3.5 and ChatGPT-4, for example, and found that 55% of works cited by GPT-3.5% and 18% of those cited by GPT-4 were fabricated (3). The authors also noted that substantive errors (authors’ names, article titles, publication dates, etc.) appeared in 43% and 24% of genuine references cited by GPT-3.5 and GPT4, respectively (3).

An investigation from 2024 (4) assessed the performance of LLMs in collecting appropriate publications for a systematic review. The researchers compared the LLM-generated sources with the references of previously published, human-conducted systematic reviews, to establish the rate of hallucinated or incorrect references provided by the LLM (4).

A recent fracas was reported (5) when the preprint server arXiv reiterated via social media (6) that their code of conduct states (7) that authors are responsible for the content of their work, regardless of how it was generated, and declared that “if a submission contains incontrovertible evidence that the authors did not check the results of LLM generation, this means we can't trust anything in the paper,” which would earn a penalty of “a 1-year ban from arXiv followed by the requirement that subsequent arXiv submissions must first be accepted at a reputable peer-reviewed venue.” (6) The message specifically mentioned hallucinated references as an example of incontrovertible evidence (6). Some submitting authors reacted with disbelief (5) that they could be held accountable for the content of the work that bore their names, including the references.

To help dispel any doubt about the importance of a properly curated reference list, in this Perspective, we will briefly discuss why your bibliography matters and what it should include. (For more detailed information on the preparation of your manuscript, please see the recommendations of the ICMJE for preparing a manuscript for submission to a medical journal (8), and specific guidelines for the target journal for your work (9). To learn about best practices in all aspects of scholarly publication, including the responsible use of AI/LLMs, see the COPE and WAME guidelines (10, 11).)

The Introduction or Background to a research paper summarizes the current evidence and presents the knowledge gap that your research is designed to address. The bibliography for your introduction provides the evidentiary foundation on which your research endeavor is based. If your references are mere hallucinations, that foundation is a house of cards. Your bibliography should show your familiarity with the current state of the science and provide the rationale for expending resources to answer your research question. Hallucinated references undermine the credibility of your work from the outset, since you have failed to establish a reason for pursuing your stated objective and aims.

The Methods section, describing patient populations studied and materials used, may require fewer references than the Introduction; however, it is necessary to provide references for established research processes, validated questionnaires, variables’ definitions as set out by professional organizations, statistical modeling approaches, and many other components of your work. Hallucinated references in your Methods section raise doubts regarding the study design, methodology, and execution, as well as regarding the replicability of your work and the validity of your results.

Your Results section may require few or no references, unless the work focuses on publications, such as reviews or bibliometric analyses. Recently, a systematic review assessed LLMs’ performance to facilitate the creation of systematic reviews (12). The authors reviewed studies evaluating LLM performance in the various tasks required in producing a systematic review, as compared to human reviewers. They found that while LLMs show promise in automating tasks such as title and abstract screening, they require human stewardship, particularly for tasks that require interpretation (12).

Perhaps the most important section of your manuscript is the Discussion. The object of the Discussion is to engage with the literature that preceded the present research and formed the foundation for your research question. In a sense, the Discussion is the author in conversation with other researchers in the field, by placing the study results in the context of the state of the art and science in the literature. Authors may show that their work supports, expands on, or controverts what has been published previously. Fabricated, inaccurate, or incomplete references in the Discussion section undermine the authors’ foundation for the reported research and its replicability, their command of the knowledge base and literature of their field, the understanding of their own results in the context of what is known, and the implications of those results for future research.

Table 1 summarizes the roles your references play in each section of your paper and the pitfalls of hallucinated sources. To avoid the hazards of hallucinated, fabricated, inaccurate, or incomplete citations, approach the proffered sources as suggestions. It is incumbent upon authors to verify reference details, for example, by searching for the book or article on its publisher or journal website or the appropriate online database (PubMed/Medline, Embase, Web of Science, etc.). Some basic tasks can be automated with dedicated tools. More importantly, authors should ensure that the content of the work is relevant to the role it will play in their own writing (Table 1). Is the work valid, has it been retracted? Does it earn its place among your bibliography?

**Table 1:** Summary of the roles of bibliography and the hazards of hallucinated references in each manuscript section.

Manuscript section	Roles of bibliography	Hazards of hallucinated references
Background	Establish the evidentiary foundation, highlight knowledge gaps, demonstrate the rationale for the research	Undermine the foundation of the research and the researcher’s knowledge of the field
Methods	Provide basis for the use of valid research tools, e.g., validated metrics and questionnaires, appropriate statistical approaches, suitable basic research methodologies	Raise doubts about the conceptual basis of the work, design of the study, and the reproducibility and internal and external validity of the results
Results	Of key importance in reviews and bibliometric research	Bad data that invalidate any analyses or conclusions
Discussion	Establish the context of your research to engage with the literature that preceded it, to demonstrate its contribution to the knowledge base, by supporting, expanding, or controverting previous work, and revealing avenues for future research	Call into question the authors’ understanding of the state of the art and science of the field along with the implications of their results in context

For guidance on the responsible use of AI in the preparation of your work for submission to Frontiers journals, please see the Guidelines for artificial intelligence and related technologies: Frontiers | Author guidelines (9) and the other resources cited above (8, 10, 11).

## Data Availability

The original contributions presented in the study are included in the article/Supplementary Material; further inquiries can be directed to the corresponding author.
